# Efficient and precise *Ultra-Quick*DASH scale measuring lymphedema impact developed using computerized adaptive testing

**DOI:** 10.1007/s11136-021-02979-y

**Published:** 2021-09-29

**Authors:** Cai Xu, Mark V. Schaverien, Joani M. Christensen, Chris J. Sidey-Gibbons

**Affiliations:** 1 MD Anderson Center for INSPiRED Cancer Care (Integrated Systems for Patient-Reported Data), The University of Texas MD Anderson Cancer Center, Houston, USA; 2 Department of Symptom Research, The University of Texas MD Anderson Cancer Center, 1515 Holcombe Blvd. Unit 1055, Houston, TX 77030-4009 USA; 3 Department of Plastic Surgery, The University of Texas MD Anderson Cancer Center, Houston, USA

**Keywords:** *Quick*DASH measure, Item response theory, Computerized adaptive testing, Lymphedema, Patient reported outcome measure, *Ultra-Quick*DASH

## Abstract

**Purpose:**

This study aimed to evaluate and improve the accuracy and efficiency of the *Quick*DASH for use in assessment of limb function in patients with upper extremity lymphedema using modern psychometric techniques.

**Method:**

We conducted confirmative factor analysis (CFA) and Mokken analysis to examine the assumption of unidimensionality for IRT model on data from 285 patients who completed the *Quick*DASH, and then fit the data to Samejima’s graded response model (GRM) and assessed the assumption of local independence of items and calibrated the item responses for CAT simulation.

**Results:**

Initial CFA and Mokken analyses demonstrated good scalability of items and unidimensionality. However, the local independence of items assumption was violated between items 9 (severity of pain) and 11 (sleeping difficulty due to pain) (Yen’s Q3 = 0.46) and disordered thresholds were evident for item 5 (cutting food). After addressing these breaches of assumptions, the re-analyzed GRM with the remaining 10 items achieved an improved fit. Simulation of CAT administration demonstrated a high correlation between scores on the CAT and the *Quick*Dash (*r* = 0.98). Items 2 (doing heavy chores) and 8 (limiting work or daily activities) were the most frequently used. The correlation among factor scores derived from the *Quick*DASH version with 11 items and the *Ultra-Quick*DASH version with items 2 and 8 was as high as 0.91.

**Conclusion:**

By administering just these two best performing *Quick*Dash items we can obtain estimates that are very similar to those obtained from the full-length *Quick*Dash without the need for CAT technology.

**Supplementary Information:**

The online version contains supplementary material available at 10.1007/s11136-021-02979-y.

## Introduction

The disabilities of the arm, shoulder, and hand (DASH) outcome measure is a widely used patient-reported outcome measure (PROM) assessing different disorders of the upper limb as well as the extent of impairments [1].

The shortened version of the DASH named the *Quick*DASH (Online Appendix A), was developed in 2005 and comprises 11 items from the original 30-item DASH while still maintaining a strong correlation with the original DASH scores [2–5]. Assessment using the *Quick*DASH takes about five minutes [6]. The use of the DASH is established for upper extremity lymphedema evaluation. Its outstanding performance in construct validity and responsiveness makes it highly recommended for breast cancer research [7, 8]. As upper extremity functioning and related activities are undoubtedly affected by the presence of lymphedema relating breast cancer treatment [9]. Compared to women without breast cancer related lymphedema, women with lymphedema have greater upper limb impairment and more movement restrictions [10]. As a measure of upper limb function, the DASH PROM has been used to measure the effect of lymphedema treatment, though it has never been specifically validated for this purpose using modern psychometric techniques [11, 12].

Modern psychometric techniques, including item response theory (IRT) and computerized adaptive testing (CAT), have been widely used to ensure that questionnaire measures are free from bias and redundant items as well as to create ‘smart’ assessments that can reduce assessment burden and increase accuracy. The *Quick*DASH was originally developed using Rasch analysis, a type of IRT analysis. The process of fitting scale data to an IRT model can identify important issues with PROMs which may interfere with the ability to derive robust and reliable scores.

The CAT is an assessment process that relies on computational algorithms to iteratively match participants to the most relevant questions for them [13]. The process of CAT can shorten legacy questionnaires as much as 82% [14]. Computerized adaptive testing synergizes with IRT insofar as it relies on the item calibration information which is derived from a successfully fitted IRT model.

In the current study, we sought to assess the advanced psychometric properties of the *Quick*DASH instrument for use in evaluation of limb function in patients with upper extremity lymphedema using IRT. Using the calibrations obtained from IRT analysis we will evaluate the performance of the *Quick*DASH when administered using CAT. We intended to also explore other options for reducing the assessment burden of the *Quick*DASH whilst still producing comparable scores with the original instrument.

## Methods

### Participants

We analyzed patient-reported outcome (PRO) data collected from 285 English speaking American adults with a diagnosis of lymphedema affecting the upper extremity in the lymphedema clinic of the University of Texas MD Anderson Cancer Center between 2016 and 2020. Mean age was 57.52 years and 197 (69.12%) were adults (< 65). The mean score for International Society of Lymphology stage was 1.98. All patients in the study had lymphedema diagnosed by measurements, including bioimpedance spectroscopy using the LDex score or limb volume using a perometer, and/or imaging, including indocyanine green (ICG) fluorescent lymphography or radionucleotide lymphoscintigraphy. The mean LDex score and limb volume difference were 22.46 and 21.73%, respectively, with cutoff thresholds of 7 for the LDex score [15–17] and 5% for limb volume measurement index used in a clinic setting for lymphedema diagnosis [18, 19].

### Measure

The *Quick*DASH PRO measures patients’ symptoms and ability to perform activities using their upper limbs during the previous week. The *Quick*DASH has 11 items scored on a 5-point Likert scale from 1 to 5 with a strong test–retest reliability [2–5, 20] and internal consistency reliability [4]. A higher item score indicates a higher level of disability or greater symptom severity [21].

### Data analysis

We assessed a series of assumptions to evaluate the scale fit to the IRT model. These assumptions included unidimensionality of the scale, scalability of items, and local independence of items. We also assessed potential issues arising from disordered items or differential item function (DIF) within the dataset. These critical terms and the corresponding mechanisms and principles behind them were shown in more details in Online Appendix B [13].

We conducted confirmatory factor analysis (CFA) with maximum-likelihood estimator to confirm the factor structure of the *Quick*DASH scale, and then assessed the fit of this model based on five main indices from the goodness of fit and residual fit statistics. We interpreted the fit of models based on relevant indicators with corresponding recommended acceptable thresholds to assist evaluation, that is, Tucker-Lewis index (TLI ≥ 0.9), comparative fit index (CFI ≥ 0.9), root mean square error of approximation (RMSEA < 0.08), root mean square of the residual (RMSR < 0.08) and a non-significant chi-square test (*p* > 0.05), however, we were mindful of type I error caused by large sample sizes in chi-square analyses [14]. We conducted Mokken analysis to further investigate the dimensional structure of the model and establish the scalability of each item [22]. Items with low scalability (Loevinger’s H < 0.30) were eliminated from further analysis [13].

We then fitted the data to Samejima’s graded response model (GRM) [23]. The GRM is suitable for developing item banks for CAT [24]. Local dependency was assessed using Yen’s Q3 with a residual correlation cut-off of + 0.20 [25]. Disordered thresholds were collapsed and rescored into adjacent categories based on proximity and logical anchor semantics. The data were re-analyzed with the eligible items left after all assumptions of IRT had been met. The fit of polytomous GRM was assessed using M2 statistics [26].

After the remaining items were calibrated to establish a bank of items using the GRM, personalizing patient assessment became possible using CAT. CAT automatically administers an item that matches the patient’s level of symptoms or functional ability based on their prior responses. In contrast to the fixed-length *Quick*DASH version, the CAT *Quick*DASH version scale can be of varied length, meaning that the specific items administered will differ from patient to patient during this adaptive testing process [27]. Specifically, the first item with the greatest information function at the distribution mean was administered by the CAT algorithm to estimate the latent trait of the lymphedema patient. After scoring based on the patient’s prior answers, the CAT algorithm will determine which is the most appropriate test question that the patient should be administered next. This estimation process will repeat until a pre-set “stopping rule” is reached. The max posterior-weighted information (MPWI) was chosen as the item selection method and the Bayesian expected a posteriori (EAP) with a prior distribution of *N* (0, 1) was used as theta estimator. The normal IRT scaling constant was set at 1.7 and the theta scale ranged from − 4 to 4. The excellent performance of these widely used parameter settings for CAT simulation with polytomous items has already been demonstrated in previous studies [28, 29]. In this study, we conducted CAT simulations for 500 repetitions each time at the stopping rule of standard errors (SE) at 0.32, 0.45, and 0.55, respectively, to explore the most efficient or precise test. As the inversed relationship between marginal reliability and SE is illustrated as reliability = 1 − SE^^2^[13], we performed three CAT simulations with different reliability of 0.9, 0.8, and 0.7 at the population mean of 0 and population standard deviation (SD) of 1.

### Software

The CFA was conducted with the “lavaan” package; the DIF detection was performed with “lordif” package; an IRT analysis was carried out with the “mokken” and “mirt” packages. The FIRESTAR code generator was adopted to simulate CAT administration [30]. All analyses were performed in the R Statistical Computing Environment [31].

## Results

### CFA

Table 1 of CFA presents the information on the item descriptive statistics and factor loading for the *Quick*DASH scale. Results show that all the factor loadings are above the cutoff point of 0.3 and loaded on the same factor, indicating adequate loadings and unidimensional structure of the *Quick*DASH PROM.

**Table 1:** Item descriptive statistics and factor loadings for the *Quick*DASH scale

Item	Mean	SD	Factor loading
Item 1	2.37 (2.37)^a^	1.11 (1.11)	0.77 (0.77)
Item 2	2.16 (2.16)	1.10 (1.10)	0.84 (0.85)
Item 3	1.83 (1.83)	0.91 (0.91)	0.80 (0.80)
Item 4	1.99 (1.99)	1.19 (1.19)	0.76 (0.76)
Item 5	1.48 (1.46)	0.91 (0.83)	0.68 (0.70)
Item 6	2.31 (2.31)	1.19 (1.19)	0.79 (0.80)
Item 7	1.68 (1.68)	1.01 (1.01)	0.75 (0.75)
Item 8	1.87 (1.87)	1.03 (1.03)	0.84 (0.84)
Item 9	2.01 (2.01)	0.99 (0.99)	0.70 (0.68)
Item 10	1.84 (1.84)	0.94 (0.94)	0.63 (0.62)
Item 11	1.66	0.90	0.65

^a^Results in parentheses are for the final round of analysis with 10 items after removing item 11

Due to the local independence issue identified in the later analysis, the finalized CFA (*χ*^2^ = 132.67, df = 35, *p* < 0.00) with 10 items substantially improved the model fit according to the goodness of fit statistics (TLI = 0.93, CFI = 0.95, RMSEA = 0.1, RMSR = 0.04),compared with the initial CFA (*χ*^2^ = 220.6, df = 44, *p* < 0.001) with 11 items (TLI = 0.89, CFI = 0.91, RMSEA = 0.12, RMSR = 0.05).

### Mokken analysis

Results from the Mokken analysis validated the unidimensional structure identified by CFA. Loevinger’s H coefficient for each item was greater than the recommended threshold for the entire scale and its constituent items (Table 2).

**Table 2:** Loevinger’s coefficient for scalability assumption test from Mokken analysis

Item	Mean	ItemH (*H*_i_)^a^	Stand Error	Dimensionality
Item 1	2.37 (2.37)^b^	0.64 (0.65)	0.03 (0.03)	1 (1)
Item 2	2.16 (2.16)	0.67 (0.69)	0.03 (0.03)	1 (1)
Item 3	1.83 (1.83)	0.65 (0.66)	0.03 (0.03)	1 (1)
Item 4	1.99 (1.99)	0.61 (0.62)	0.03 (0.03)	1 (1)
Item 5	1.48 (1.46)	0.60 (0.62)	0.05 (0.04)	1 (1)
Item 6	2.31 (2.31)	0.64 (0.65)	0.03 (0.03)	1 (1)
Item 7	1.68 (1.68)	0.61 (0.62)	0.04 (0.04)	1 (1)
Item 8	1.87 (1.87)	0.68 (0.68)	0.03 (0.03)	1 (1)
Item 9	2.01 (2.01)	0.59 (0.57)	0.04 (0.04)	1 (1)
Item 10	1.84 (1.84)	0.53 (0.53)	0.04 (0.04)	1 (1)
Item 11	1.66	0.55	0.04	1

^a^Scale H for initial round analysis with11 items and final round analysis with 10 items are 0.62 (0.03) and 0.63 (0.03), respectively

^b^Results for the final round of analysis including 10 items are in parentheses

### Graded response model

As unidimensionality and scalability assumptions for the IRT model were met through CFA and Mokken analyses, and no DIF items were detected in the groups of adults and older adults (≥ 65), the GRM based on the IRT framework was conducted using all 11 items. The estimated parameters of discriminations (*a*) and difficulty (*b*) are presented in Table 3 and utilized to illustrate the relationship between the overall disability level and the corresponding item. To facilitate the interpretation, these parameters were transformed into *Z*-scores with a mean of 0 and an SD of 1. The assumption of Local independence of items was assessed within the GRM. The largest residual correlation among item 9 (severity of pain) and item 11 (sleeping difficulty due to pain) (Yen’s Q3 = 0.46) was above the acceptable threshold of + 0.2 [32]. Item 11 (sleeping difficulty due to pain) was therefore removed from further analysis completely.

**Table 3:** Discrimination and difficulty parameter estimates for the *Quick*DASH scale

Item	*a*	*b*1	*b*2	*b*3	*b*4	Factor 1
Item 1	2.45 (2.47)^a^	− 0.89 (− 0.89)	0.27 (0.27)	1.24 (1.24)	2.05 (2.04)	0.82 (0.82)
Item 2	3.44 (3.51)	− 0.48 (− 0.48)	0.45 (0.45)	1.31 (1.31)	2.08 (2.08)	0.90 (0.90)
Item 3	2.96 (3.01)	− 0.19 (− 0.18)	0.91 (0.91)	2.03 (2.02)	2.75 (2.74)	0.87 (0.87)
Item 4	2.24 (2.25)	− 0.15 (− 0.15)	0.70 (0.70)	1.49 (1.49)	2.11 (2.10)	0.80 (0.80)
Item 5	2.30 (2.32)	0.69 (0.69)	1.37 (1.37)	2.23 (2.23)	2.65	0.80 (0.81)
Item 6	2.64 (2.68)	− 0.57 (− 0.57)	0.28 (0.27)	1.24 (1.23)	1.93 (1.92)	0.84 (0.84)
Item 7	2.45 (2.46)	0.28 (0.28)	1.09 (1.09)	1.81 (1.80)	2.49 (2.48)	0.82 (0.82)
Item 8	3.36 (3.34)	− 0.09 (− 0.09)	0.79 (0.79)	1.54 (1.53)	2.56 (2.56)	0.89 (0.89)
Item 9	2.06 (1.93)	− 0.40 (− 0.41)	0.65 (0.67)	1.94 (1.99)	3.02 (3.12)	0.77 (0.75)
Item 10	1.47 (1.43)	− 0.27 (− 0.27)	1.24 (1.25)	2.34 (2.37)	3.84 (3.91)	0.65 (0.64)
Item 11	1.81	0.16	1.35	2.37	3.34	0.73

^a^Results for the final round analysis including 10 items are in parentheses

**Fig. 1:**
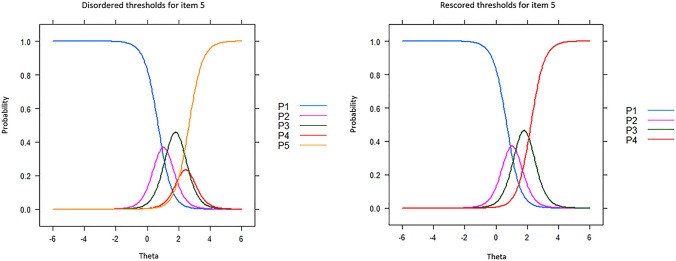
Collapsing thresholds for item 5 “Rating your ability of using a knife to cut food in the last week” (Recoded item 5 goes with 1-2-3-4-4 instead of 1-2-3-4-5 after accounting for the disordered response category thresholds 4 and 5)

Additionally, initial GRM analysis with 11 items detected the issue of disordered response categories for item 5 (cutting food) based on its item characteristic curve. And the new rescored item 5 (cutting food) with 4 response categories is displayed in Fig. 1.

The GRM was re-analyzed with the remaining 10 items. Results of the residual correlation indicated the assumption of local independence of items had been reasonably met. Estimated parameters for the 10 items also are shown in parentheses in Table 3. All the 10 items left had a strong level of discrimination (*a*), indicating they are better at distinguishing between patients at specific disability levels.

The fit of the GRM to data were also evaluated through item fit and model fit assessment. Results indicated that the remaining 10 items reasonably fit the model (*p* > 0.05) and the model fit the data well based on the goodness of fit index (TLI = 0.88, CFI = 0.96, RMSEA = 0.09, RMSR = 0.05). Hence, the GRM adequately fit to the data set (see Online Appendix C).

**Fig. 2:**
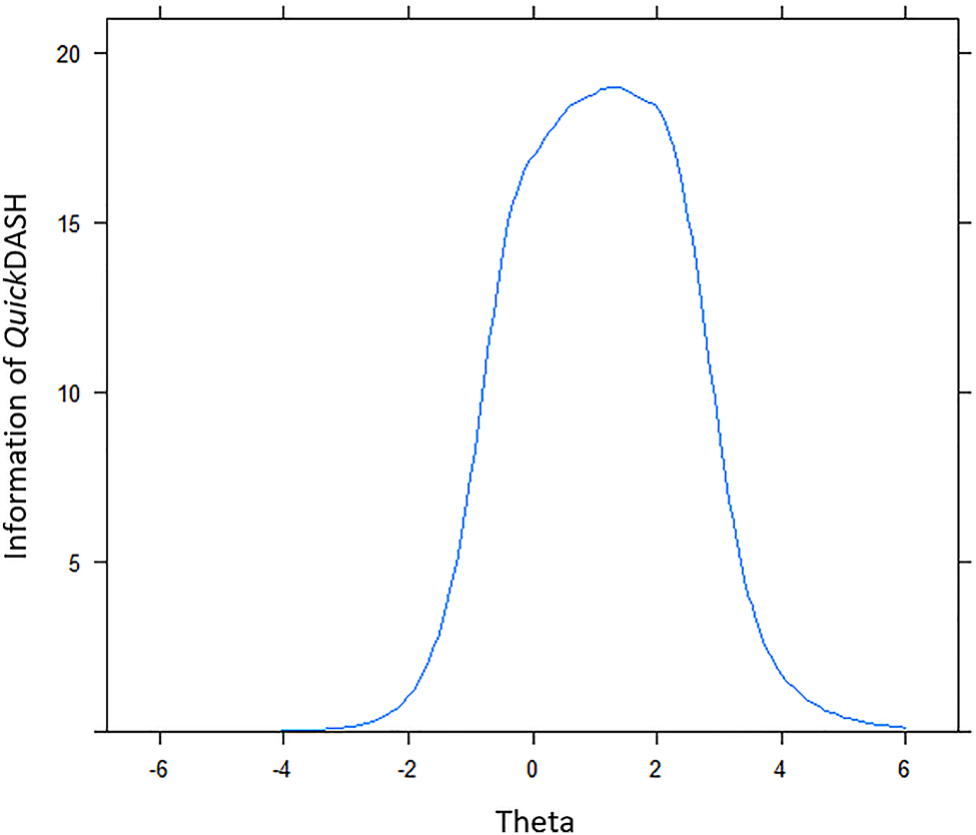
Test information curve of the *Quick*DASH scale with 10 items

The test information curve of the GRM with the remaining 10 items (Fig. 2), calculated by accumulating each item information together, showed that the entire *Quick*DASH instrument provides much more information for respondents with a higher level of disability symptom due to the peak of the curve is located above the average theta (*θ*) 0. The latent trait of patients with disability symptoms above the average theta level (*θ*) 0 will be precisely estimated through this instrument.

### CAT simulation

The results of the average number of items used for each time, the correlation between thetas (*θ*), mean SE, item mean, item media, item range are summarized in Table 4. During the 500 iterations, there 78 participants with SEs were higher than the pre-specified SE of 0.32.

**Table 4:** Results of three times *Quick*DASH CAT simulations with varied SEs

	SE (0.32)	SE (0.45)	SE (0.55)
Alpha (*α*)	.90	.80	.70
Average number of items used	3.36	3.06	2
Correlation between thetas	0.98	0.97	0.96
mean SE^a^	0.32	0.34	0.35
Item Mean	3.36	3.06	2
Item median	2	2	2
Item SD^b^	2.68	2.65	0
Item range	2–10	2–10	2–2
Time of iterations	500	500	500

^a^SE = standard error

^b^SD = standard deviation

**Fig. 3:**
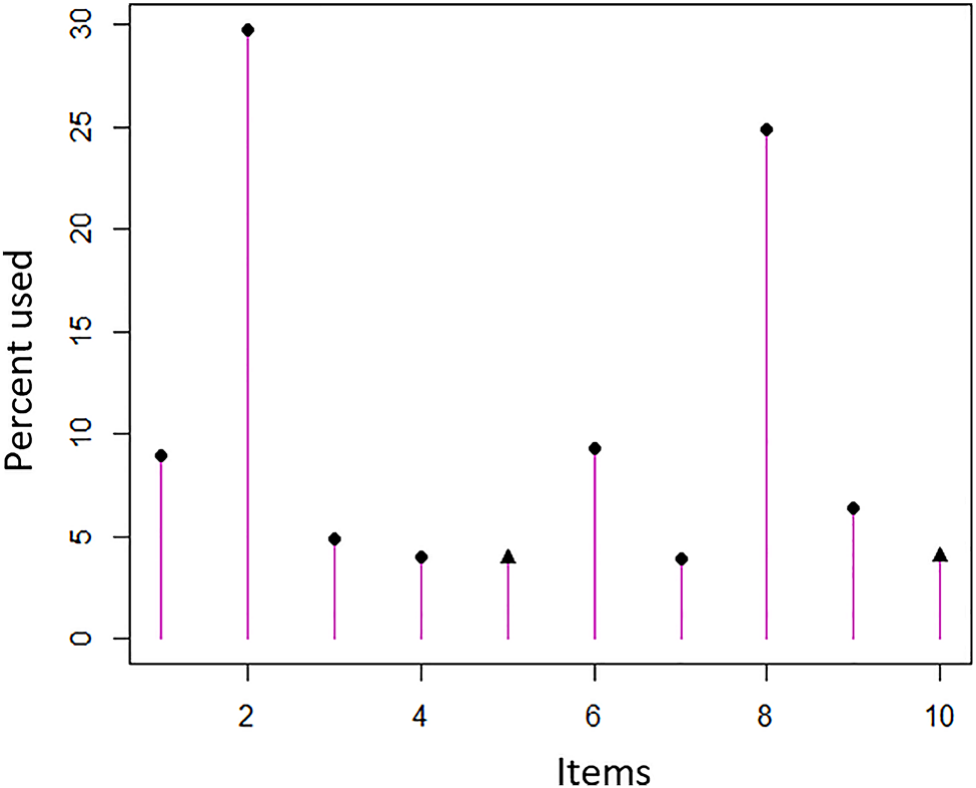
Frequency of items used in the *Quick*DASH CAT simulation

Among them, item 2 (doing heavy chores) and item 8 (limiting work or daily activities) were most exposed during the CAT simulation due to their more item information providing (Fig. 3). Table 5 shows that 72.78% of the information provided by Items 2 and 8 were centered on the theta range of (− 2, + 2). The estimates of the level of *Quick*DASH trait score provided by the simulated CAT algorithm and the original *Quick*DASH trait score derived from the fixed-length questionnaire correlated highly up to 0.98 with mean score of − 0.01 (SD = 0.97), 0.97, and 0.96, respectively.

**Table 5:** Item information provided in specified range of full *Quick*DASH

Item	Specified range	Information provided for specified range (%)	Total information provided for the whole scale
All 11 items	(− 10, + 10)	73.03 (100%)	73.03
All 11 items	(− 2, + 2)	48.62 (66.58%)	73.03
Item 2	(− 2, + 2)	7.75 (77.69%)	9.98
Item 8	(− 2, + 2)	6.82 (67.89%)	10.05
Items 2 and 8	(− 2, + 2)	14.57 (72.78%)	20.02

### Comparison among full *Quick*DASH, CAT, and *Ultra-Quick*DASH

**Fig. 4:**
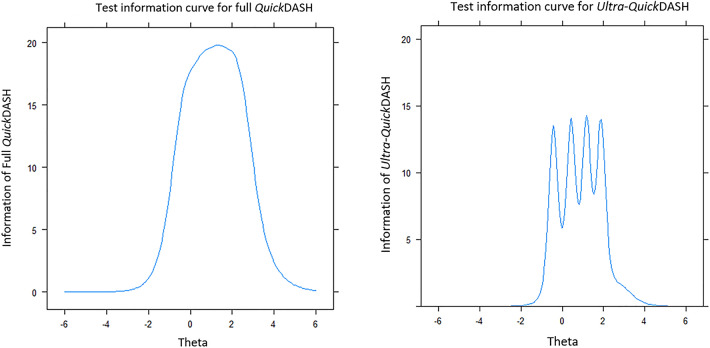
Test information curves for full *Quick*DASH with 11 items and *Ultra-Quick*DASH with 2 items

Tables 6 and 7 present the comparison results of participant score among these three versions of established DASH. Full *Quick*DASH had the highest mean participant score of 0.001 (SD = 0.96); the root mean square deviation (RMSD = 0.19) and SD of difference (0.19) between CAT and full *Quick*DASH comparison were lower. Both full *Quick*DASH and *Ultra*-*Quick*DASH provided much more information for participants with disabilities above the average level (*θ*) in Fig. 4.

**Table 6:** Basic information of full *Quick*DASH, CAT, and *Ultra*-*Quick*DASH

DASH version	Included item (n)	Participant score				
		Mean	SD	Min	Max	Median
*Quick*DASH	Items 1–11 (11)	0.001	0.96	− 1.67	2.80	− 0.02
CAT^a^	Items 1–10 (10)	− 0.01	0.97	− 1.52	2.85	0.16
*Ultra*-*Quick*DASH	Items 2, 8 (2)	− 0.0003	0.92	− 1.12	2.48	0.08

^a^ Results are from CAT 500 simulation with a stopping rule of SE = 0.32

**Table 7:** Comparison of participant scores among full *Quick*DASH, CAT, and *Ultra*-*Quick*DASH

	Correlation between participant scores	Mean difference	SD^a^ of difference	RMSD^b^
*Ultra*-*Quick*DASH vs *Quick*DASH	0.90	− 0.001	0.41	0.41
CAT vs *Quick*DASH	0.98	− 0.09	0.19	0.19

^a^SD = Standard deviation

^b^RMSD = Root mean square deviation

## Discussion

### Principal findings

We showed that the *Quick*DASH could be made to fit the IRT model, with minor modification, for evaluation of limb function in patients with upper extremity lymphedema. Once fitted to the IRT model we demonstrated that the assessment length can be dramatically reduced without sacrificing assessment accuracy using either CAT or a 2-item short form. The demonstrated strengths of the CAT approach in improving efficiency and precision in this study are consistent with the findings in previous studies [13, 14].

Studying only an American sample substantially exempts the study from the issues of DIF resulting from cultural diversity and hence provides a suitable item bank to be used within the US society setting. Cronbach alpha value (*α* = 0.93) from CFA indicated the excellent internal consistency in the entire *Quick*DASH scale. However, after applying the GRM to fit the data, the Yen’s Q3 value showed that items 9 (severity of pain) and 11(sleeping difficulty due to pain) violated the assumption of local independence of items. As many other reasons can attribute difficulty in sleeping, this may explain the reduced item information in item 11 (sleeping difficulty due to pain) compared to item 9 (severity of pain).

The excellent performance of the CAT simulation with the remaining 10 items provides useful information for the *Quick*DASH instrument in clinical practice. It is foreseeable that the developed CAT *Quick*DASH, as a supporting instrument for healthcare providers’ decision making regarding individual patient care, not only reduces the burden of patient-reported assessment and facilitates quick data collection and storage but also further promotes the enforceability and actionability of feedback and ultimately benefits patients with all upper extremity disorders from improved health care and research.

Also, we note that items 2 (doing heavy chores) and 8 (limiting work or daily activities) were most frequently administered during the CAT simulation. As the ability to do heavy labor may invoke swelling for lymphedema patients and activities of daily living were most affected by lymphedema [33], this reasonably explains why these two highly discriminative items dominated the information of the *Quick*DASH scale. The two items that dominated the CAT administration suggest that a reasonable ultra-short and technology-free version of *Quick*DASH can be developed only including items 2 and 8. Furthermore, results indicate that the correlation among the factor score of level of disability for lymphedema patients calculated from the *Quick*DASH including 11 items, and the *Ultra-Quick*DASH version only containing these two best items, was exceptionally high (*r* = 0.90). In this way, it is more operable for these health institutions or clinics that are not familiar with the CAT algorithm as they can use a super shorter and more accurate *Ultra-Quick*DASH questionnaire.

### Limitations

This study comes with several limitations. *First*, negative results of residual correlation between items come out from the assumption of local independence of items test revealed the possibility of multidimensional structure of the *Quick*DASH data, which just provides one plausible explanation for slightly high RMSEA (0.09) for the *Quick*DASH scale with 10 items. Further research is warranted to investigate the reason behind this and refine the *Quick*DASH instrument. *Second*, to address the local dependency issue, we removed the item with lower item information from further analysis directly and have not compared with the other widely used method of collapsing the items into a testlet on the possible influence on the analysis results [14]. *Third*, the analysis results are based on the data collected from the MD Anderson Cancer Center institute only. Additional data from other clinical centers are needed to externally validate, even update these findings, and further promote the application of *Ultra-Quick*DASH into clinic practices widely. *Fourth*, a cross-cultural adaptive test of different language versions of the *Ultra-Quick*DASH scale on measuring disability and symptoms related to lymphedema needs to be conducted in future research although DIF is not applicable in this study. *Fifth*, the relatively small number of items used to calibrate the item bank will slightly affect the precision of underlying construct estimation during CAT simulation [14]. *Sixth*, the sample size is relatively small (*n* = 285), however, the distribution of DASH score (factor score of disability) was normal with acceptable skewness (0.28) and kurtosis (− 0.34) [34], which may suggest that item parameters will be stable in larger populations. Seventh, we wish to caution users that the reduced length version inevitably will exclude some relevant questions from participants. While we demonstrate that this has a limited impact on DASH scores at the population level, it is foreseeable that some individual scores may differ substantially between the full-length DASH and both the CAT and fixed-length *Ultra*-*Quick*DASH versions. Additionally, The *Ultra*-*Quick*DASH provides less information on assessment participants than the complete *Quick*DASH and is not recommended in a situation where assessment reliability should be prioritized over brevity.

### Conclusion

By utilizing CAT simulation based on the IRT framework to shorten the *Quick*DASH substantially, we found that a more efficient and precise estimation of disability level and symptom severity for American lymphedema patients can be achieved. In the meanwhile, the Concerto, as an emerging open-source CAT delivery platform, makes this CAT *Quick*DASH application in a real clinic setting possible [35]. All the improvements achieved will facilitate the PROM development and ultimately improve the health care and research to benefit patients. Moreover, the developed *Ultra-Quick*DASH mainly consisting of two best performing items and maintaining efficient and accurate estimations could be used as a CAT technology-free version of *Quick*DASH. Its application and promotion can break the obstacles of complex technology on health care professionals and providers on the use of CAT *Quick*DASH, making this super shortened instrument more convenient to apply into routine clinic practices.

## Supplementary Information

Below is the link to the electronic supplementary material.

Supplementary file1 *Quick*DASH Questionnaire (PDF 676 kb)

Supplementary file2 (PDF 690 kb)

Supplementary file3 Revised *Quick*DASH Questionnaire (PDF 213 kb)

## Data Availability

Available on request.

## Code availability

Available on request.
