# Supplementary material for: Efficient and precise Ultra-QuickDASH scale measuring lymphedema impact developed using computerized adaptive testing
Source: Qual Life Res. 2021 Sep 29;31(3):917–25. doi: 10.1007/s11136-021-02979-y (PMC8921172; doi:10.1007/s11136-021-02979-y)
Supplement: Supplementary file 1 — Supplementary file1 QuickDASH Questionnaire (PDF 676 kb) [file 11136_2021_2979_MOESM1_ESM.pdf]

Appendix A

Efficient and Precise *Ultra-Quick*DASH Scale Measuring Lymphedema Impact Developed Using Computerized Adaptive Testing

(to be submitted to the *Quality of Life Research Journal*)

Cai Xu[1,2] • Mark V. Schaverien[3] • Joani M. Christensen[3] • Chris J. Sidey-Gibbons[1,2*]

[1]MD Anderson Center for INSPiRED Cancer Care (Integrated Systems for Patient-Reported Data), The University of Texas MD Anderson Cancer Center, Houston, USA

[2]Department of Symptom Research, The University of Texas MD Anderson Cancer Center, Houston, USA

[3]Department of Plastic Surgery, The University of Texas MD Anderson Cancer Center, Houston, USA

* **Corresponding author**

Prof. Chris Sidey-Gibbons, PhD

Email: cgibbons@mdanderson.org   Office Mobile: (713) 598-3674

The University of Texas, MD Anderson Cancer Center, Symptom Research CAO, 1515 Holcombe Blvd. Unit 1055, Houston, TX 77030-4009

THE *Quick*DASH

OUTCOME MEASURE

INSTRUCTIONS

This questionnaire asks about your symptoms as well as your ability to perform certain activities.

Please answer *every question*, based on your condition in the last week, by circling the appropriate number.

If you did not have the opportunity to perform an activity in the past week, please make your *best estimate* of which response would be the most accurate.

It doesn't matter which hand or arm you use to perform the activity; please answer based on your ability regardless of how you perform the task.

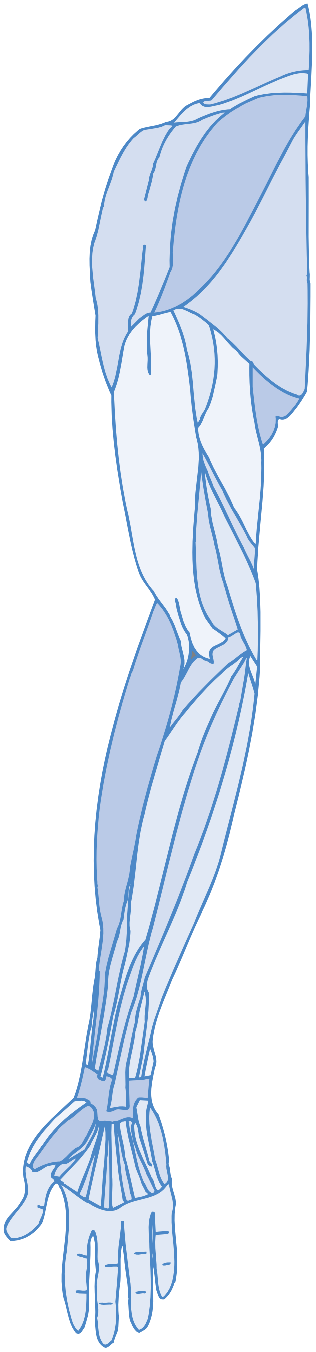

NAME ________________________________

DATE ____________________

MR No. ___________________

*Quick*DASH

Please rate your ability to do the following activities in the last week by circling the number below the appropriate response.

	NO DIFFICULTY	MILD DIFFICULTY	MODERATE DIFFICULTY	SEVERE DIFFICULTY	UNABLE
1. Open a tight or new jar.	1	2	3	4	5
2. Do heavy household chores (e.g., wash walls, floors).	1	2	3	4	5
3. Carry a shopping bag or briefcase.	1	2	3	4	5
4. Wash your back.	1	2	3	4	5
5. Use a knife to cut food.	1	2	3	4	5
6. Recreational activities in which you take some force or impact through your arm, shoulder or hand (e.g., golf, hammering, tennis, etc.).	1	2	3	4	5

	NOT AT ALL	SLIGHTLY	MODERATELY	QUITE A BIT	EXTREMELY
7. During the past week, *to what extent* has your arm, shoulder or hand problem interfered with your normal social activities with family, friends, neighbours or groups?	1	2	3	4	5

	NOT LIMITED AT ALL	SLIGHTLY LIMITED	MODERATELY LIMITED	VERY LIMITED	UNABLE
8. During the past week, were you limited in your work or other regular daily activities as a result of your arm, shoulder or hand problem?	1	2	3	4	5

**Please rate the severity of the following symptoms in the last week.** *(circle number)*	NONE	MILD	MODERATE	SEVERE	EXTREME
9. Arm, shoulder or hand pain.	1	2	3	4	5
10. Tingling (pins and needles) in your arm, shoulder or hand.	1	2	3	4	5

	NO DIFFICULTY	MILD DIFFICULTY	MODERATE DIFFICULTY	SEVERE DIFFICULTY	SO MUCH DIFFICULTY THAT I CAN'T SLEEP
11. During the past week, how much difficulty have you had sleeping because of the pain in your arm, shoulder or hand? *(circle number)*	1	2	3	4	5

***Quick*DASH DISABILITY/SYMPTOM SCORE** = $\left(\left[\frac{\text{sum of n responses}}{\text{n}}\right] - 1\right) \times 25$, where n is equal to the number of completed responses.

A *Quick*DASH score may not be calculated if there is greater than 1 missing item.
