# Supplementary material for: Efficient and precise Ultra-QuickDASH scale measuring lymphedema impact developed using computerized adaptive testing
Source: Qual Life Res. 2021 Sep 29;31(3):917–25. doi: 10.1007/s11136-021-02979-y (PMC8921172; doi:10.1007/s11136-021-02979-y)
Supplement: Supplementary file 2 — Supplementary file2 (PDF 690 kb) [file 11136_2021_2979_MOESM2_ESM.pdf]

Appendix B

Efficient and Precise *Ultra-Quick*DASH Scale Measuring Lymphedema Impact Developed Using Computerized Adaptive Testing

(to be submitted to the *Quality of Life Research Journal*)

Cai Xu[1,2] • Mark V. Schaverien[3] • Joani M. Christensen[3]• Chris J. Sidey-Gibbons[1,2*]

[1]MD Anderson Center for INSPiRED Cancer Care (Integrated Systems for Patient-Reported Data), The University of Texas MD Anderson Cancer Center, Houston, USA

[2]Department of Symptom Research, The University of Texas MD Anderson Cancer Center, Houston, USA

[3]Department of Plastic Surgery, The University of Texas MD Anderson Cancer Center, Houston, USA

* **Corresponding author**

Prof. Chris Sidey-Gibbons, PhD

Email: cgibbons@mdanderson.org   Office Mobile: (713) 598-3674

The University of Texas, MD Anderson Cancer Center, Symptom Research CAO, 1515 Holcombe Blvd. Unit 1055, Houston, TX 77030-4009

Critical Terms and Corresponding Mechanisms of IRT Analysis

Unidimensionality of scale

Unidimensionality of the scale assumption refers only one dimension or a single metric is measured by this scale being studied. Otherwise, the measure will be uninterpretable if it embraces more than one dimension. We can use factor analysis to ascertain the dimensionality such as exploratory factor analysis (EFA) or confirmatory factor analysis (CFA), the utilization preference of which relies on whether the underlying structure of the scale is established or not. Addition, Monkken analysis is an alternative to further verify the dimensionality explored by factor analysis [1].

Scalability of items

Scalability of items ensures we gain the interval level measurement and monotonically increasing item response function. We assessed the scalability of items based on Loevinger's H coefficient generated from Mokken analysis [2]. Items or the scale were considered to obtain sufficient scalability only when the Loevinger's H reached 0.30 or above [3].

Graded response model (GRM)

The graded response model (GRM) [4] as a flexible and polytomous-response IRT model was employed for the data. The characteristics of varied discriminations among items, and unchanged functional form when merging response categories, and being easy to understand make it far superior to one parameter (e.g. Rasch model) [5] and two-parameter models (e.g. generalized partial credit model) [6]. Discrimination (a) and difficulty (b) and were produced within the GRM analysis. The former parameter examines the difficulty level of each item when a test-taker has a 50% probability to endorse the latent trait; the latter parameter reflects how good an item is to discriminate between respondents on the different level of the underlying trait.

Local independence of items

Local independence of items means that items of the scale should be uncorrelated after controlling for the latent variable, which is assessed with Yen's Q3 value for correlations between item residuals. Item residual correlation of more than 0.2 indicates the breach of local independence between items assumption [7]. High residuals correlation leans to occur when items that are too similar and lead to inflating reliability and model misfit [8]. So far, three ways are available to address this issue if the Yen's Q3 is larger than 0.2, that is, deleting this item directly based on sound grounds, retaining items but only administer one of them into the analysis, or adding both to a testlet.

Category threshold ordering

Polytomous response with 5 response categories was scored on a 5 Likert scale from 1 to 5 for each item in this study. Ordered categories mean that the categories are modal, otherwise the overall model fit will be negatively affected. Category threshold ordering was also examined by viewing item characteristic curves to ensure the interval level measurement and guarantee each category is utilized in the same way for respondents. Disordered thresholds will be collapsed and rescored to maintain the right ordering.

Differential item function (DIF)

Differentiative item function (DIF) hypothesizes that the scores on the patient-reported outcome measurement (PROM) should not change because of the demographic group [9]. DIF occurs when different groups have a different probability of endorsing the specific items, even though they are detected to have the same level of ability. The bias caused by DIF could reduce validity for between-group comparisons and bring greater impact to the CAT due to the limited number of items to be administered. Deleting and ignoring these DIF items are current practices to address this issue [10]. However, if more than 50% items are detected as DIF items, separate scales are suggested for these individual groups [11].

References

1. Sijtsma, K., Emons, W. H. M., Bouwmeester, S., Nyklíček, I., & Roorda, L. D. (2008). Nonparametric IRT analysis of Quality-of-Life Scales and its application to the World Health Organization Quality-of-Life Scale (WHOQOL-Bref). *Quality of Life Research*, *17*(2), 275–290. https://doi.org/10.1007/s11136-007-9281-6

2. Stochl, J., Jones, P. B., & Croudace, T. J. (2012). Mokken scale analysis of mental health and well-being questionnaire item responses: A non-parametric IRT method in empirical research for applied health researchers. *BMC Medical Research Methodology*, *12*, 74. https://doi.org/10.1186/1471-2288-12-74

3. Gibbons, C., Bower, P., Lovell, K., Valderas, J., & Skevington, S. (2016). Electronic Quality of Life Assessment Using Computer-Adaptive Testing. *Journal of Medical Internet Research*, *18*(9), e240. https://doi.org/10.2196/jmir.6053

4. Samejima, F. (1969). Estimation of latent ability using a response pattern of graded scores. *Psychometrika Monograph Supplement*, *17*(4), 2. https://doi.org/doi:10.1002/j.2333-8504.1968.tb00153.x

5. Thissen, D., & Orlando, M. (2001). Item response theory for items scored in two categories. In D.Thissen & H.Wainer (Eds.), *Test scoring* (pp. 73–140). Mahwah, NJ:Lawrence Erlbaum Associates Publishes.

6. Reeve, B. B., Hays, R. D., Bjorner, J. B., Cook, K. F., Crane, P. K., Teresi, J. A., Thissen, D., Revicki, D. A., Weiss, D. J., Hambleton, R. K., Liu, H., Gershon, R., Reise, S. P., Lai, J. S., & Cella, D. (2007). Psychometric evaluation and calibration of health-related quality of life item banks: Plans for the Patient-Reported Outcomes Measurement Information System (PROMIS). *Medical Care*, *45*(5 SUPPL. 1). https://doi.org/10.1097/01.mlr.0000250483.85507.04

7. Yen, W. M. (1993). Scaling Performance Assessments: Strategies for Managing Local Item Dependence. *Journal of Educational Measurement*, *30*(3), 187–213. https://doi.org/10.1111/j.1745-3984.1993.tb00423.x

8. Wright, B. D. (1996). Local dependency, correlations and principal components. *Rasch Measurement Transactions*, *10*(3), 509–511.

9. Tennant, A., Penta, M., Tesio, L., Grimby, G., Thonnard, J. L., Slade, A., Lawton, G., Simone, A., Carter, J., Lundgren-Nilsson, A., Tripolski, M., Ring, H., Biering-Sørensen, F., Marincek, C., Burger, H., & Phillips, S. (2004). Assessing and adjusting for cross-cultural validity of impairment and activity limitation scales through differential item functioning within the framework of the Rasch model: the PRO-ESOR project. *Medical Care*, *42*(1 Suppl). https://doi.org/10.1097/01.mlr.0000103529.63132.77

10. Cho, S. J., Suh, Y., & Lee, W. Y. (2016). After Differential Item Functioning Is Detected: IRT

Item Calibration and Scoring in the Presence of DIF. *Applied Psychological Measurement*, *40*(8), 573–591. https://doi.org/10.1177/0146621616664304

11. Bolt, D. M., Vitale, J. E., Hare, R. D., & Newman, J. P. (2004). A multigroup item response theory analysis of the psychopathy checklist - Revised. In *Psychological Assessment* (Vol. 16, Issue 2, pp. 155–168). https://doi.org/10.1037/1040-3590.16.2.155
