# Supplementary material for: Efficient and precise Ultra-QuickDASH scale measuring lymphedema impact developed using computerized adaptive testing
Source: Qual Life Res. 2021 Sep 29;31(3):917–25. doi: 10.1007/s11136-021-02979-y (PMC8921172; doi:10.1007/s11136-021-02979-y)
Supplement: Supplementary file 3 — Supplementary file3 Revised QuickDASH Questionnaire (PDF 213 kb) [file 11136_2021_2979_MOESM3_ESM.pdf]

Revised *Quick* DASH

Please rate your ability to do the following activities in the last week by circling the number below the appropriate response.

	**NO DIFFICULTY**	**MILD DIFFICULTY**	**MODERATE DIFFICULTY**	**SEVERE DIFFICULTY**	**UNABLE**
1. Open a tight or new jar.	1	2	3	4	5
2. Do heavy household chores (e.g., wash walls, floors).	1	2	3	4	5
3. Carry a shopping bag or briefcase.	1	2	3	4	5
4. Wash your back.	1	2	3	4	5
5. Use a knife to cut food.	1	2	3	4	4
6. Recreational activities in which you take some force or impact through your arm, shoulder or hand (e.g., golf, hammering, tennis, etc.).	1	2	3	4	5
	**NOT AT ALL**	**SLIGHTLY**	**MODERATELY**	**QUITE A BIT**	**EXTREMELY**
7. During the past week, to what extent has your arm, shoulder or hand problem interfered with your normal social activities with family, friends, neighbours or groups?	1	2	3	4	5
	**NOT LIMITED AT ALL**	**SLIGHTLY LIMITED**	**MODERATELY LIMITED**	**VERY LIMITED**	**UNABLE**
8. During the past week, were you limited in your work or other regular daily activities as a result of your arm, shoulder or hand problem?	1	2	3	4	5
**Please rate the severity of the following symptoms in the last week**. *(circle number)*	**NONE**	**MILD**	**MODERATE**	**SEVERE**	**EXTREME**
9. Arm, shoulder or hand pain.	1	2	3	4	5
10. Tingling (pins and needles) in your arm, shoulder or hand.	1	2	3	4	5

*Quick***DASH DISABILITY/SYMPTOM SCORE**= $\left(\left[\frac{\text{(sum of n responses)}}{n}\right]-1\right)^{x25,}$ where n is equal to the number of completed responses.

A *Quick*DASH score may <u>not</u> be calculated if there is greater than 1 missing item.
